# Survey dataset on the impact of stakeholder's relationship on the academic performance of engineering students

**DOI:** 10.1016/j.dib.2018.02.059

**Published:** 2018-02-27

**Authors:** Opeyemi Oyeyipo, Henry Odeyinka, James Owolabi, Adedeji Afolabi, Rapheal Ojelabi

**Affiliations:** a Covenant University, Ota, Ogun State, Nigeria; b Obafemi Awolowo University, Ile-Ife, Osun State, Nigeria

**Keywords:** Academic performance, Stakeholder's relationship, Lecturer–student relationship, University

## Abstract

In order to produce seasoned graduates from tertiary institutions, academic performance of students should be paramount in the minds of stakeholders. The dataset presented the perception of engineering students and lecturers in two private universities in Ogun state, namely, Bells University of Technology and Covenant University. Purposive quota sampling was used to elicit data from students and lecturers in the institutions through a closed ended structured questionnaire. Inferential statistics such as component principal analysis, regression analysis and Kruskall Wallis test were used to present the data. The engineering students are in their fourth year. The data collected focused on stakeholder's relationship on students’ academic performance. It also provided information on the significant factors affecting stakeholder's relationship in tertiary educational institution as well as the effect of the age of the students in lecturer–student relationship. The survey data when analysed can be a pointer in identifying the unique stakeholders’ characteristics that could engender best academic performance from the students.

**Specifications Table**

**Table:** 

Subject area	*Social science*
More specific subject area	*Relationship Management*
Type of data	*Tables and Figures*
How data was acquired	*Field Survey*
Data format	*Raw*
Experimental factors	*Purposive sampling of engineering students and lecturers in two (2) tertiary institutions*
Experimental features	*First descriptive statistics were provided, component principal analysis and Kruskall Wallis were prepared. Then a multivariate regression was performed in three stages, testing the correlations between center-periphery price gradients and immigrant populations, as well as other socio-economic features.*
Data source location	*Lagos and Ogun State, Nigeria*
Data accessibility	*The data are attached to this article*

.

**Value of the data**

• The data were collected from the two major stakeholders-students and lecturers in academic environment and also provides the role of age of students in determining the lecturer–student's relationship in academic environment.
• The dataset presented provides original indicator of the effect of stakeholders’ relationship on engineering students’ academic performance in selected Universities in Nigeria.
• This is the largest dataset available on the impact of stakeholders’ relationship in universities in the country.
• The dataset can be used to identify the significant factors affecting stakeholders’ relationship in universities.
• When the unique characteristics of lecturers’ are carefully understood from the data provided, the influence on the academic performance can be detected.

## 1 Data

**Fig. 1:**
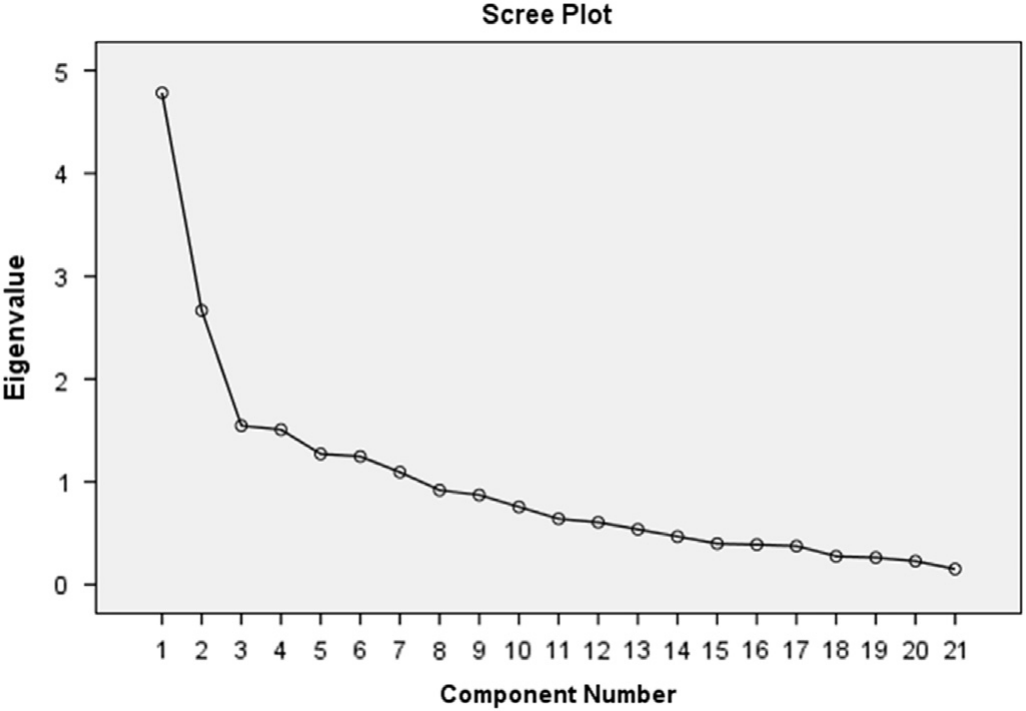
Scree Plot of factors affecting stakeholders’ relationship.

**Fig. 2:**
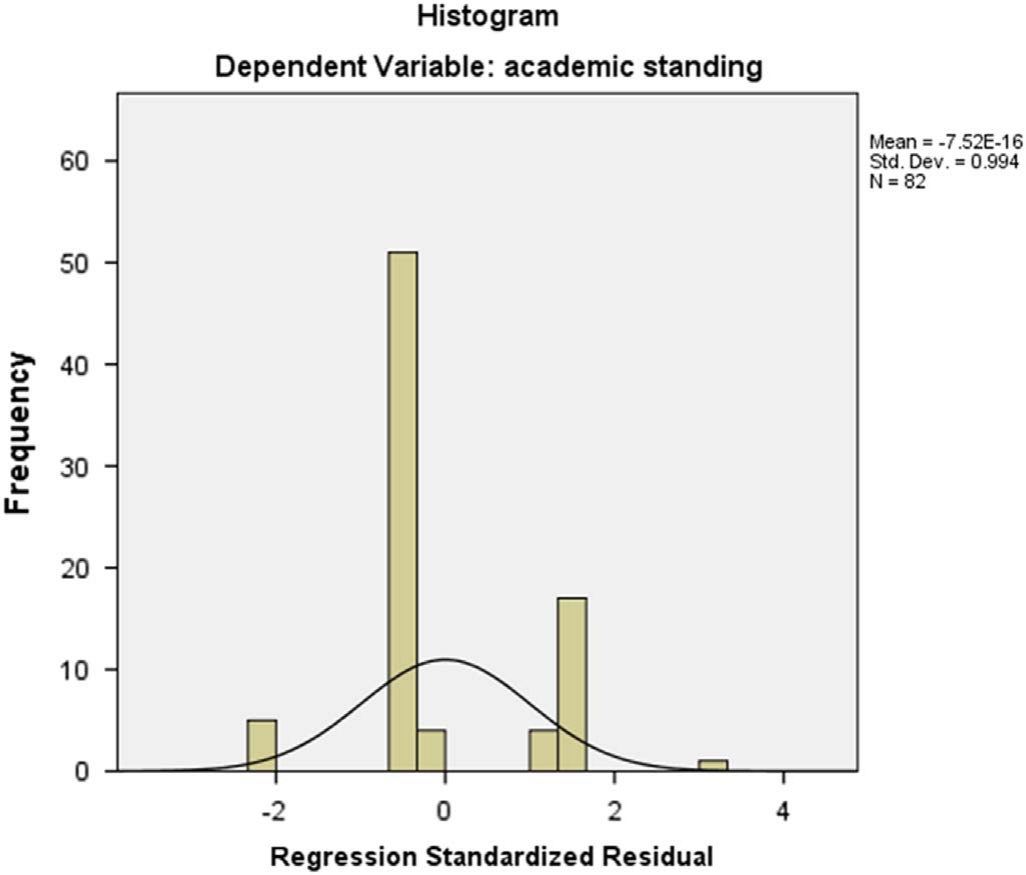
Histogram of the regression impact of stakeholder's relationship on academic performance.

The dataset presented was collected from fourth year engineering students and their lecturers from two (2) renowned private universities in Ogun state, Nigeria on stakeholders’ relationship and academic performance of students. The distribution of students/lecturers from the two institutions are shown in Table 1. The lecturers and fourth year engineering students in the two prominent private universities in the state were the target respondents. Majority of the target respondents participated in the survey, however after scrutinizing the data instrument for errors and inconsistency, 210 questionnaire were returned for analysis. The designed data instrument elicited information on factors affecting stakeholders’ relationship and the influence it has on the academic performance of engineering students. Furthermore, the influence of the characteristics of age of the student on the lecturer–student relationship was tested. For tertiary institutions to succeed, there is need to focus on issues that can engender quality academic performance of their students. By understanding this data, the contributions of the lecturer characteristics as it may influence student performance can be easily dissected from the data. In order to group the factors affecting stakeholders’ relationship, component principal analysis was used. Table 2 shows that the KMO measure for sampling adequacy was 0.782, which is larger than 0.7, suggesting that the sample was acceptable for factor analysis. The Bartlett's test was 548.260 and the associated significance level was *p*-value < 0.001, indicating that the population correlation matrix was not an identity matrix. Both of the tests showed that the obtained data supported the use of factor analysis. Cronbach's Alpha of 0.758 suggested that the reliability of the data instrument used was also acceptable. Fig. 1 shows the scree plat of the variables, which showed a breakage at the third variable depicting that there are three (3) main groups to the factors affecting stakeholders’ relationship. Table 3, Table 4 lists the eigenvalues associated with each linear component before extraction, after extraction and after rotation. The data investigated the impact of the stakeholders’ relationship on academic performance of students. Table 5 and Fig. 2 show the Regression analysis and the Histogram of the regression test of the impact of stakeholder's relationship on academic performance respectively. The data tested further the influence of age of the student on the lecturer–student relationship in the tertiary institutions. The data is presented in Table 6. The data obtained when analysed can be used as a comparative study with students in other faculties/college. The influence of other stakeholders apart from lecturers can be explored further.

**Table 1:** Distribution of fourth year students from the selected universities for the study.

Faculties/Institution	**Covenant University**	**Bells University of Technology**
Students	113	70
Lecturer	15	12
Total	128	82

**Table 2:** KMO and Bartlett's Test of factors affecting stakeholders’ relationship.

Kaiser–Meyer–Olkin Measure of Sampling Adequacy	0.782
Bartlett's Test of Sphericity:	
Approx. Chi-square	548.260
Degree of freedom	210
Significant level	0.000

**Table 3:** Total Variance Explained (Eigenvalues) of factors affecting stakeholders’ relationship.

Component	Initial Eigenvalues		
	Total	Percent of Variance	Cumulative Percentage of Variance
1	4.784	22.779	22.779
2	2.666	12.696	35.476
3	1.546	7.364	42.840
4	1.508	7.179	50.019
5	1.272	6.056	56.075
6	1.247	5.936	62.011
7	1.094	5.209	67.220

**Table 4:** Rotated Component Matrix factors affecting stakeholders’ relationship.

	Component		
	1	2	3
Personality traits of students	0.750		
Students’ sense of belonging	0.711		
Reluctance of students to approach lecturers	0.641		
Experience of the lecturers	0.522		
Lecturers’ style and method of lecturing	0.514		
School's rules and regulations about students and lecturers interrelationships	0.482		
Availability and accessibility of lecturers	0.479		
Personality traits of lecturers	0.478		
Students’ lack of interest in academic activities	0.454		
Incessant closure of school as a result of strikes and students’ unionism	0.452		
Family background of the students		0.733	
Family responsibilities of the lecturers		0.682	
Religious differences between students and lecturer		0.670	
Gender differences between students and lecturers		0.623	
Differences in Cultural norms between students and lecturers		0.550	
Lecturers’ manner of behaving and reacting to suggestions from students.			0.731
Stress and burn-out of lecturers as a result of academic workload			0.638
Students’ manner of behaving and reacting to instructions from lecturers			0.636
Age differences between students and lecturers			0.551
Students’ inexperience in managing their lecturers			0.404

**Table 5:** Regression of the impact of stakeholder's relationship on academic performance.

	*B*	Std. Error	*t*	*p* value
(Constant)	2.388	0.392	6.092	0.000
**Student lecturer relationship**	0.048	0.111	0.435	0.664
Model’ Summary				
*R* = 0.049				
*R*^2^ = 0.002				
Adjusted *R*^2^ = 0.010				

Dependent variable; academic standing; Std error- Standard error; *B*= Unstandardized co-efficient; *p* value= significance value

**Table 6:** Kruskall Wallis showing age of student's effect on student lecturer relationship.

	**Student lecturer relationship**
Chi-square	2.512
df	3
Asymp.Sig	0.473

Grouping variable: age category

## 2 Experimental design, materials and methods

The two (2) private universities in Ogun state selected for the study accounted for the renowned institutions within the state. Bells university of Technology is the first private technology institutions while Covenant University is one of the best universities in the country. The research instrument considered demographic variables such as; age of the respondents, status amongst others. The study highlighted twenty-one (21) factors affecting stakeholders’ relationship in universities from literature [1], [2], [3], [4], [5], [6]. In the same vein, the study identified six (6) variables of student–lecturer relationships peculiar to the Nigerian University system [1], [2], [3], [4]. The population comprised of lecturers and students in engineering fields in areas under consideration. A purposive sampling technique was used in selecting the sample size. A cross-sectional survey design using a questionnaire instrument was used to elicit data from the respondents. Similar works that have used field survey instrument to obtain data can be found in works by [7], [8], [9], [10], [11], [12], [13], [14], [15], [16], [17], [18], [19]. The questionnaire was divided into three parts; demographic variables, factors affecting stakeholders’ relationship and lecturer–student relationship in academic settings. The measurement scale for majority of the research instrument is ordinal scale. The data was analyzed using the Statistical Package for Social Science (SPSS) software. Outstanding academic performance should be paramount in the survival of tertiary institutions. In order to churn out quality students from higher institutions, studies should focus on different factors that can engender exceptional performance from the students. The dataset is useful for lecturers to understand the qualities they possess in order to enhance the academic performance of the students they are tutoring.
