# Supplementary material for: Survey dataset on the impact of stakeholder's relationship on the academic performance of engineering students
Source: Data Brief. 2018 Feb 27;17:1355–60. doi: 10.1016/j.dib.2018.02.059 (PMC5854871; doi:10.1016/j.dib.2018.02.059)
Supplement: Supplementary file 1 — Supplementary material [file mmc1.docx]

CONFLICT OF INTEREST

THERE EXIST NO CONFLICT OF INTEREST AS THE INCLUSION OF REFERENCE WAS THE ONLY TALKING POINT TILL DATE
